# Isolation measures and protection awareness are significant for latent tuberculosis infection: a cross-sectional study based on T-SPOT.*TB* among health care workers in China

**DOI:** 10.1017/S0950268818002777

**Published:** 2019-03-11

**Authors:** Yunfeng Deng, Yun Liu, Yan Li, Hui Jing, Yan Wang, Xuezheng Li, Lingzhong Xu

**Affiliations:** 1 Department of Social Medicine and Health Service Management, School of Public Health, Shandong University, Jinan, Shandong, 250012, China; 2 Katharine Hsu International Research Center of Human Infectious Diseases, Shandong Provincial Chest Hospital Affiliated to Shandong University, Jinan, Shandong, 250013, China; 3 Department of Clinical Laboratory, Shandong Provincial Hospital Affiliated to Shandong University, Jinan, Shandong, 250021, China; 4 Department of Infection Control, Shandong Provincial Chest Hospital Affiliated to Shandong University, Jinan, Shandong, 250013, China

**Keywords:** Different working locations or job categories, health care workers, latent tuberculosis infection, risk factors, T-SPOT.*TB* assay

## Abstract

This study aimed to reveal the associated risk factors for latent tuberculosis infection (LTBI) detected by T-SPOT.*TB* assay among health care workers (HCWs) at different working locations or job categories in China. This cross-sectional study included 934 HCWs who underwent the T-SPOT.*TB* assay. Demographic and social characteristics of the participants, including age, sex, job categories, department/ward and duration of healthcare service, were recorded. Among 934 HCWs, 267 (28.5867%) were diagnosed as having LTBI with positive T-SPOT.*TB* assay. HCWs working in inpatient tuberculosis (TB) (odds ratio (OR) 2.917; 95% confidence interval (CI) 1.852–4.596; *P* < 0.001) and respiratory wards (OR 1.840; 95% CI 1.124–3.011; *P* = 0.015), and with longer duration of healthcare service (OR 1.048; 95% CI 1.016–1.080; *P* = 0.003) were risk factors for positive T-SPOT.*TB* result. Furthermore, longer working duration increased the positive rate of T-SPOT.*TB* results for physicians and nurses, and physicians had higher risks than nurses for the same working duration. Inpatient TB and respiratory wards were high-risk working locations for HCWs with LTBI, and longer duration of healthcare service also increased the risk of LTBI among HCWs. A complete strategy for TB infection control and protection awareness among HCWs should be enhanced.

## Introduction

Tuberculosis (TB) infection remains a threat to global public health security [1], and can infect the lungs, lymphatic system and other organs [2]. As one of the countries with high TB burden, China has been reported to have an incidence of 67/100 000 population per year, and the number of incident cases of TB in China ranks third worldwide [3]. Although great efforts have been made, China still faces a huge burden on the quality of life of TB patients [4].

In general, health care workers (HCWs) are considered to have a higher risk for TB infection because of more frequent exposure to patients with active TB compared with the general population [5–7]. The detailed guidelines of latent TB infection (LTBI) screening and preventive therapy have been implemented for HCWs of high-income countries, and a clear decline in TB infections have been demonstrated under serial surveillance [8]. However, the risk of TB infection is more serious in HCWs of low- and middle-income countries, with an annual LTBI incidence of 5.8% and a high LTBI prevalence ranging from 33% to 79% [9]. Thus, a rigorous TB control programme for HCWs is very necessary in low- and middle-income countries.

Previous studies have reported the risk factors for LTBI in HCWs, such as advanced age, male, smoking history, duration of healthcare service, job category, delayed diagnosis, misdiagnosis and lacking or inadequate personal protective measures [10–12]. Due to the low cost and simple technology, the tuberculin skin test (TST) is widely used for determining LTBI [13]. Unfortunately, the non-tuberculous mycobacteria and *Mycobacterium bovis* bacillus Calmette–Guerin (BCG) vaccine strain cannot be differentiated and identified by TST, leading to poor specificity and high false-positive reactions [14, 15]. Interferon *γ* release assays (IGRAs) are also more specific tools for diagnosing LTBI [16]. The enzyme-linked immunospot assay (ELISPOT) (T-SPOT.*TB*, Oxford Immunotec Ltd., Abingdon, Oxon, UK) based on IGRAs has been considered as a new tool to diagnose LTBI [16]. Several studies have shown that HCWs at different working locations or job categories may have different risks for TB infection [17, 18]. However, there were limited systematic studies investigating the relationship between working locations or job categories and risk of LTBI based on the T-SPOT.*TB* assay in HCWs in China.

The aim of this study was to evaluate and analyse the risk factors for LTBI among HCWs at different working locations or job categories in Shandong Provincial Chest Hospital (SPCH) by LTBI screening using T-SPOT.*TB* assay and to analyse the changes in T-SPOT.*TB* results from 2010 to 2016 in HCWs. This study may provide valuable evidence for the establishment of TB control programmes for HCWs.

## Methods

### Study design and participants

All of the HCWs at SPCH were recruited in this cross-sectional study. The inclusion criteria were: (1) HCWs who were tested by the T-SPOT.*TB* assay in 2016; and (2) the complete basic information and test results were recorded. The exclusion criteria were: (1) HCWs with TB history; (2) pregnant and lactating women; and (3) HCWs who refused to participate in this study. This study was approved by the Ethics Committee of Shandong Province Chest Hospital.

#### Setting

SPCH is a provincial specialised lung and heart hospital with 1020 certified HCWs and has a bed capacity of 931, including 381 TB care beds (88 beds with negative pressure facilities), 550 beds for respiratory, tumour, cardiovascular and other departments. Based on hospital management categorisation, the working location is classified as follows: (1) general medicine facility: department of medical administration and nursing department; (2) inpatient TB ward: patients diagnosed as pulmonary TB and extrapulmonary infection, as well as patients with negative result of sputum smear culture; (3) laboratory facility; (4) outpatient medicine; (5) respiratory ward; (6) negative pressure ward for smear-positive and/or culture-positive TB patients; and (7) inpatient non-TB ward. The job categories of the hospital included (1) administrative staff, including the management and finance; (2) clinical officers from the clinical management department, medical administration department, nursing department, the office of quality control, the office of hospital infection management, the office of medical and health care service; (3) physician; (4) laboratory technician from clinical laboratory and pathology department; (5) nurse; (6) patient attendant of medical imaging department, X-ray, B-type ultrasonography; and (7) ward attendant.

#### Data collection and T-SPOT.*TB* assay

Demographic and social characteristics of participants, including age, sex, job categories, department/ward and duration of healthcare service, were recorded. Blood specimen was collected from each HCW for the T-SPOT.*TB* assay. T-SPOT.*TB* assay was performed using T-SPOT.*TB* assay kit (Oxford Immunotec Ltd.) according to the manufacturer's instructions. Briefly, peripheral blood mononuclear cells were extracted from the blood specimens of participants, followed by washing and concentration. The concentration of cells was diluted to 2.5 × 10^6^ cells/mL. The cells were seeded onto pre-coated plates and then incubated with two antigens (ESAT6 and CFP10) or positive control for 16–20 h at 37 °C. The cells in the culture plates were washed using phosphate-buffered saline four times, and then the chromogenic substrate solution was added to each well for 7 min at room temperature. Finally, the number of spots was calculated using ELISPOT automatic plate reader (Oxford Immunotec Ltd.). The results were determined according to the following algorithm: positive: ⩾6 spots and (2) negative: ⩽5 spots. LTBI was diagnosed as a positive T-SPOT.*TB* result.

#### Statistical analysis

Statistical analysis was performed using SPSS 18.0 software (SPSS Inc., Chicago, IL, USA). Measurement data were expressed as the mean ± standard deviation, and analysed using independent samples *t-*test. Enumeration data were represented as *n* (%) and were analysed using *χ*^2^ test. Demographic and social variables related to LTBI were included in multiple logistic regression model used to analyse the risk factors of LTBI. A *P*-value of <0.05 was considered statistically significant.

## Results

### Participant characteristics

There were 1020 HCWs in 2016 at this hospital, among whom 934 HCWs (713 females and 221 males, mean age of 36.15 ± 9.42 years) met inclusion criteria and were included in this study. The mean duration of healthcare service was 11.99 ± 9.47 years. Of the 934 HCWs, 267 (28.5867%) were diagnosed as LTBI with positive result for T-SPOT.*TB* assay and other HCWs were negative for the test.

### The risk factors for LTBI in HCWs

**Fig. 1.**
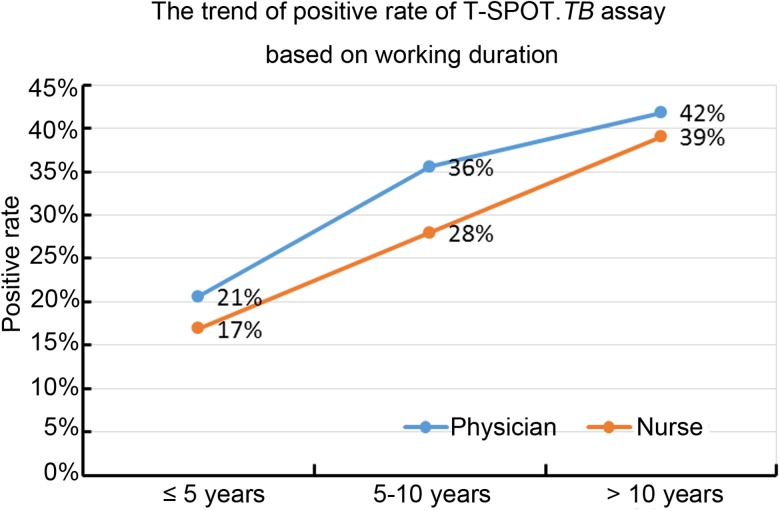
The positive rate of T-SPOT.*TB* assay in physicians and nurses with different working duration.

Results of the univariate analysis (Table 1) showed that the rate of positive T-SPOT.*TB* assay was only closely associated with older age (*P* < 0.001), longer duration of healthcare service (*P* < 0.001) and working location (*P* = 0.002). The inpatient TB ward (41.9118%) and the respiratory ward (30.8333%) had higher risk of LTBI than the negative pressure ward (20.383%). Further multivariable analysis (Table 2) demonstrated that HCWs working in the inpatient TB ward (odds ratio (OR) 2.917; 95% confidence interval (CI) 1.852–4.596; *P* < 0.001) and the respiratory ward (OR 1.840; 95% CI 1.124–3.011; *P* = 0.015), as well as longer duration of healthcare service (OR 1.048; 95% CI 1.016–1.080; *P* = 0.003) were risk factors for LTBI. Moreover, the results revealed that the positive rate of T-SPOT.*TB* assay increased in both physicians and nurses when their working duration increased; moreover, physicians had higher positive rate than nurses for the same working duration (Fig. 1).

**Table 1.** Univariate analysis of positive T-SPOT.*TB* results

Factors		*N*	Positive results	Negative results	*P*-value
Age, years (mean, SD)		934	38.37 ± 9.63	35.26 ± 9.20	0.000
Sex (*n*, %)					
Male		221	69 (31.2217)	152 (68.7783)	0.321
Female		713	198 (27.7700)	515 (72.2300)	
Duration of healthcare service, years (mean, SD)		934	14.73 ± 10.07	10.89 ± 9.00	0.000
Working location (*n*, %)					
General medicine facility		331	87 (26.2840)	244 (73.7160)	0.002
Inpatient TB ward		136	57 (41.9118)	79 (58.0882)	
Inpatient non-TB ward		161	39 (24.2236)	122 (75.7764)	
Laboratory facility		36	8 (22.2222)	28 (77.7778)	
Outpatient medicine		47	18 (38.2979)	29 (61.7021)	
Respiratory ward		120	37 (30.8333)	83 (69.1667)	
Negative pressure ward		103	21 (20.3883)	82 (79.6117)	
Job categories (*n*, %)					
Administrative staff		106	23 (21.6981)	83 (78.3019)	0.307
Clinical officers		51	14 (27.4510)	37 (72.5490)	
Physicians		148	51 (34.4595)	97 (65.5405)	
Laboratory technicians		36	8 (22.2222)	28 (77.7778)	
Nurses		450	127 (28.2222)	323 (71.7778)	
Patient attendants		61	45 (73.7705)	16 (26.2295)	
Ward attendants		82	28 (34.1463)	54 (65.8537)	

SD, standard deviation; TB, tuberculosis

**Table 2.** Multivariate analysis of factors associated with positive T-SPOT.*TB* results

Factors	OR	95% CI	*P*-value
General medicine facility			
Inpatient TB ward	2.917	1.852–4.596	0.000
Inpatient non-TB ward	1.291	0.809–2.061	0.284
Laboratory facility	1.022	0.438–2.388	0.960
Outpatient medicine	1.655	0.861–3.182	0.131
Respiratory ward	1.840	1.124–3.011	0.015
Negative pressure ward	1.079	0.613–1.900	0.791
Age	1.003	0.972–1.035	0.845
Duration of healthcare service	1.048	1.016–1.080	0.003

TB, tuberculosis; OR, odds ratio; CI, confidence interval.

### The changes of T-SPOT.*TB* results from 2010 to 2016 in HCWs

A total of 476 HCWs had T-SPOT.*TB* results in both 2010 and 2016. The T-SPOT.*TB* results showed that the conversion rate (from negative in 2010 to positive in 2016) was 24.7899% (118/476) in these HCWs at different working locations and job categories, while the reversion rate (from positive in 2010 to negative in 2016) was 3.9916% (19/476) (Table 3). Based on different working locations, the reversion rates were 8.4746% and 9.0909% in the inpatient TB ward and the negative pressure ward, respectively, which were higher than other working locations. On the other hand, the conversion rates in the inpatient TB ward (38.9830%) and the respiratory ward (30.3571%) were higher than other wards. In addition, high reversion rates among clinical officers (6.8965%) and ward attendants (5.7692%) were found, while the conversion rates were increased among clinical officers (31.0345%), physicians (28.3582%) and nurses (27.4039%). However, the change in the rates of T-SPOT.*TB* results had no significant statistical differences among different working locations as well as different job categories (Table 3).

**Table 3.** Conversion (from negative in 2010 to positive in 2016), reversion (from positive in 2010 to negative in 2016) or no change in rates between 2010 and 2016 of T-SPOT.*TB* results based on different working locations and job categories

Factors	Results			*P*-value
	Conversion rate	Reversion rate	No change rate	
Working locations (*n*, %)				
General medicine facility	7 (3.4483)	41 (20.1970)	155 (76.3547)	0.063
Inpatient TB ward	5 (8.4746)	23 (38.9830)	31 (52.5424)	
Inpatient non-TB ward	2 (2.7397)	18 (24.6575)	53 (72.6028)	
Laboratory facility	1 (4.3478)	5 (21.7391)	17 (73.9131)	
Outpatient medicine	1 (3.4483)	8 (27.5862)	20 (68.9655)	
Respiratory ward	0 (0)	17 (30.3571)	39 (69.6429)	
Negative pressure ward	3 (9.0909)	6 (18.1818)	24 (72.7273)	
Job categories (*n*, %)				
Administrative staff	0 (0)	9 (13.8460)	56 (86.1538)	0.282
Clinical officers	2 (6.8965)	9 (31.0345)	18 (62.0690)	
Physicians	3 (4.4776)	19 (28.3582)	45 (67.1642)	
Laboratory technicians	1 (4.3478)	5 (21.7391)	17 (73.9131)	
Nurses	9 (4.3269)	57 (27.4039)	142 (68.2692)	
Patient attendants	1 (3.1250)	7 (21.8750)	24 (75.0000)	
Ward attendants	3 (5.7692)	12 (23.0769)	37 (71.1539)	
Total (*n*, %)	19 (3.9916)	118 (24.7899)	339 (71.2185)	

TB, tuberculosis.

## Discussion

It is well known that HCWs are more likely to acquire TB than the general population; thus, the huge efforts on TB infection control are urgent. The present study found that among 934 HCWs, positive T-SPOT.*TB* results were found in 28.5867% of HCWs. The risk factors for LTBI among HCWs at different working locations or job categories were then investigated, which may contribute to the establishment of TB infection control programmes for HCW.

Several epidemiological studies have been conducted to compare the prevalence of LTBI detected by TST and IGRAs in HCWs, and have shown 72% agreement between TST and IGRAs [19]. A recent study has demonstrated that the prevalence of LTBI in HCWs was 31.5% and 25.0% based on TST and IGRAs, respectively, in a moderate TB burden country [20]. As a high TB burden country, the study in China has revealed that the prevalence of LTBI detected by TST in HCWs was 58.0% in an infectious diseases hospital and 33.9% in a non-TB hospital [21]. In addition, Zhou *et al*. have shown that in a general hospital in Beijing (China), 55.2% of HCWs were positive by TST and 28.7% of cases were positive by T-SPOT.*TB* [22]. Similarly, our study revealed a prevalence of 28.5867% for LTBI detected by T-SPOT.*TB* in HCWs. Notably, the prevalence of LTBI in HCWs was 9.9% in Germany [23] and 6.0% in the USA by T-SPOT.*TB* [13]. These studies suggest that compared with developed countries, developing countries have a higher prevalence of LTBI in HCWs, and the lower prevalence in developed countries may be due to the rigorous implementation and surveillance of LTBI screening and prevention. Thus, TB control programmes for HCWs should be implemented more rigorously to reduce LTBI in China.

In this study, the results of the T-SPOT.*TB* assay revealed that HCWs with advanced age was associated with positive T-SPOT.*TB* result, which is consistent with previous studies that have also reported higher prevalence of LTBI in older HCWs [24–27]. A possible reason for this result is that older HCWs have worked in a hospital for a sufficiently long time to expose themselves to a TB environment in comparison with younger HCWs. Supportively, the present study identified longer duration of healthcare service as an independent risk factor for LTBI with positive T-SPOT.*TB* result. Consistent with our study, a previous study has reported that the prevalence of LTBI in HCWs is closely related to longer duration of employment, and HCWs with 10 years or more of employment have higher risk for LTBI than those with <1 year of employment [28]. Recent studies have also demonstrated that being employed for longer time increases the risk of LTBI in HCWs [24, 26]. In addition, our study found that the positive rate of T-SPOT.*TB* assay was positively related to longer working duration in physicians and nurses, and physicians had higher risk than nurses when they had the same working duration. This can be explained that nurse teams have a higher awareness of TB infection control and personal protection in real clinic practice, and they receive management guidance and training more often than physician teams.

Furthermore, the current study revealed that the working locations of HCWs were closely associated with the risk of LTBI, and the inpatient TB ward and the respiratory ward were considered as risk factors for high prevalence of LTBI. This result is consistent with a previous report, and it had shown that HCWs who worked in a ward related to TB patients have a higher risk of LTBI [27]. Notably, our study also found that no significant difference was found in the changes of T-SPOT.*TB* results from 2010 to 2016 based on different working locations and job categories. Moreover, the conversion rates (from negative in 2010 to positive in 2016) of the T-SPOT.*TB* assay were high in the inpatient TB ward and the respiratory ward, as well as among clinical officers, physicians and nurses; and reversion rates (from negative in 2010 to positive in 2016) were high in the inpatient TB ward and the negative pressure ward, as well as among clinical officers and ward attendants It is well known that TB spreads easily through the air. Although the guidelines on isolation of and protection from TB infection in HCWs have been recommended by the WHO and China's health administration, the special isolation and protection measures cannot be implemented well in practice, such as at SPCH. The number of negative pressure wards is insufficient to treat patients with TB infection in SPCH. Only smear-positive or/and culture-positive TB patients are treated in negative pressure wards, which leads to more TB patients being admitted to general wards. Moreover, due to imperfect laboratory testing techniques, patients with active TB cannot be accurately and fully diagnosed, which also results in more TB patients in general wards, and even in the respiratory ward. As shown in this study, HCWs in the negative pressure ward had lower risk of LTBI than in the inpatient TB ward and the respiratory ward. Therefore, although the diagnostic level has improved in China, sub-optimal isolation measures and protection awareness may be responsible for the high prevalence of LTBI in HCWs.

Our study had several limitations. First, this was a retrospective study, and the results from this study should be confirmed in a future prospective study. Second, in this study, HCWs were recruited from SPCH with more patients with TB; therefore, these results may not reflect the comprehensive situation in tertiary hospitals. Third, there was lack of investigation about awareness of education and knowledge of TB prevention and control. Finally, because of the low TB incidence of Shandong province in China, our observations may not be generalised to other provinces with high incidence in China.

In conclusion, our data reveal that a longer duration of healthcare service is an independent risk factor for LTBI among HCWs in China. Furthermore, the inpatient TB and respiratory wards have a higher risk for LTBI among HCWs than the negative pressure ward, and the inpatient TB and respiratory wards were also identified as independent risk factors for LTBI. These results prompted that the lack of isolation measures and protection awareness of HCWs greatly influence the high risk of LTBI. Therefore, the current strategy for TB infection control and protection awareness among HCWs should be enhanced.
